# Molecular and Functional Analysis of TLR 1, 2 and 6 in Peripheral Blood Monocytes of Patients with Schizophrenia: A Pilot Study

**DOI:** 10.3390/ijms26030926

**Published:** 2025-01-23

**Authors:** Carlo E. Sotelo-Ramírez, Marcela Valdés-Tovar, Julio Uriel Zaragoza-Hoyos, Leonardo Ortiz-López, Jesús Argueta, Mauricio Rosel-Vales, Roxana U. Miranda-Labra, Beatriz Camarena

**Affiliations:** 1 Doctorado en Biología Experimental, Universidad Autónoma Metropolitana (UAM)-Iztapalapa, Mexico City 09340, Mexico; csotelor9494@gmail.com; 2 Departamento de Farmacogenética, Subdirección de Investigaciones Clínicas, Instituto Nacional de Psiquiatría Ramón de la Fuente Muñiz, Mexico City 14370, Mexico; biojuzh@hotmail.com; 3 Subdirección de Investigaciones Clínicas, Instituto Nacional de Psiquiatría Ramón de la Fuente Muñiz, Mexico City 14370, Mexico; mvaldes@inprf.gob.mx (M.V.-T.); leosan@inprf.gob.mx (L.O.-L.); 4 Laboratorio de Neurofarmacología, Subdirección de Investigaciones Clínicas, Instituto Nacional de Psiquiatría Ramón de la Fuente Muñiz, Mexico City 14370, Mexico; jadclear@yahoo.com; 5 Dirección de Servicios Clínicos, Instituto Nacional de Psiquiatría Ramón de la Fuente Muñiz, Mexico City 14370, Mexico; 6 Departamento de Ciencias de la Salud, Universidad Autónoma Metropolitana (UAM)-Iztapalapa, Mexico City 09340, Mexico

**Keywords:** TLR1, TLR2, TLR6, single-nucleotide polymorphisms, gene expression, schizophrenia

## Abstract

Schizophrenia (SZ) is a chronic disabling mental disorder with high heritability, and several immune-regulating genes have been implicated in its pathophysiology In this study, we investigated the expression of Toll-like receptors (TLRs) 1, 2, and 6 in peripheral blood monocytes from SZ patients and healthy control subjects (HCSs) in the Mexican population, focusing on specific SZ-associated gene variants. Gene expressions were assessed by qPCR, and protein expression was measured using flow cytometry. The secretory profiles of MALP2-stimulated monocytes were evaluated through immunoproteomic arrays. Our results indicate that patients with SZ carrying the rs4833093/*TLR1* GG genotype exhibited significantly lower *TLR1* gene expression compared to TT carriers. Notably, HCSs with the TT genotype showed markedly higher *TLR1* protein expression, while all patients with SZ exhibited significantly reduced protein levels regardless of genotype. Furthermore, monocytes from patients with SZ displayed altered secretion profiles upon TLR stimulation, with significant elevations in IL-18, uPAR, angiopoietin-2, and serpin E1, alongside reductions in MCP-1, IL-17A, IL-24, MIF, and myeloperoxidase compared to HCSs. These findings suggest a dysfunctional TLR-mediated innate immune response in SZ.

## 1. Introduction

Schizophrenia (SZ) is considered a disabling, complex, and chronic psychiatric disorder characterized by five symptomatic domains, which can include positive, negative, aggressive, depressive, and cognitive symptoms [1]. According to the World Health Organization, SZ affects over 21 million people worldwide [2]. Family, twin, and adoption studies have indicated that the predisposition to develop SZ is among the highest for psychiatric disorders, with an estimated heritability of approximately 0.81, highlighting a significant genetic component in its etiology [3]. Genome-wide association studies (GWASs) support a relationship between regulatory genes of the immune response and SZ [4]. Two of the largest GWASs to date [5,6] identified associations with regions within the major histocompatibility complex (MHC), which contains over 200 genes that are key regulators of immune function [7,8]. Post-GWAS analyses have further implicated biological pathways associated with glutamatergic transmission, the gut–brain axis, cytokine signaling, and Toll-like receptor (TLR) signaling [9].

Toll-like receptors (TLRs) are a primary family of pattern recognition receptors that initiate innate immune response. Upon the recognition of pathogen- or damage-associated molecular patterns (PAMPs or DAMPs, respectively), TLRs activate pathways that lead to the synthesis of pro-inflammatory molecules [9]. In preclinical models, mice lacking the *tlr2* gene have exhibited behavioral alterations that resemble the negative symptoms of SZ [10]. Altered levels of biochemical mediators, including cytokines and chemokines, have also been reported in patients with SZ [11,12,13]. Additionally, postmortem brain analyses of patients with SZ reveal an altered gene expression of molecules critical to the TLR signaling cascade, such as myeloid differentiation primary response gene 88 (MyD88) and nuclear factor-κB (NF-κB) [14]. Furthermore, Kozłowska et al. [15] reported alterations in the expression of different TLRs in European patients with SZ compared with a control group. A previous genetic association study in Mexican population identified that the rs4833093/*TLR1* variant confers an increased risk of developing SZ, while the rs5743709/*TLR2* and rs3775073/*TLR6* variants provide protection [16]. Also, a GxG interaction analysis showed an increased risk of developing SZ between the *TLR1*/rs483309 and *TLR2*/rs5743709 variants [16]. This evidence suggests that TLRs could be related to SZ; however, the effect of the rs4833093, rs5743709, and rs3775073 gene variants on the expression and function of their corresponding receptors is yet unknown.

Taking this into consideration, the aims of this study were to (1) determine the effect of rs483309 of the *TLR1*, rs5743709 of the *TLR2*, and rs3775073 of the *TLR6* by the evaluation of mRNA and protein expression levels in monocytes of patients with SZ and HCSs, and (2) evaluate the TLR2/TLR6 heterodimer function by the analysis of the ligand-stimulated secretory profile of monocytes of patients with SZ and HCSs.

## 2. Results

### 2.1. Demographic and Clinical Characteristics of the Sample

The clinical and demographic characteristics of the sample are summarized in Table 1. There were no significant differences in age and sex between patients and controls. Regarding substance use, neither patients nor controls reported illegal drug consumption. Therefore, only tobacco and alcohol consumption in patients and controls are presented in Table 1, showing no significant differences between the groups. Patients had a higher percentage of single individuals than controls. Patients were more likely to have only basic education, while controls showed a higher frequency of advanced education levels. Similarly, a higher proportion of unemployed individuals were noted among patients compared to controls.

### 2.2. Immunofluorescent Detection of CD14, TLR1, TLR2 and TLR6

**Figure 1:**
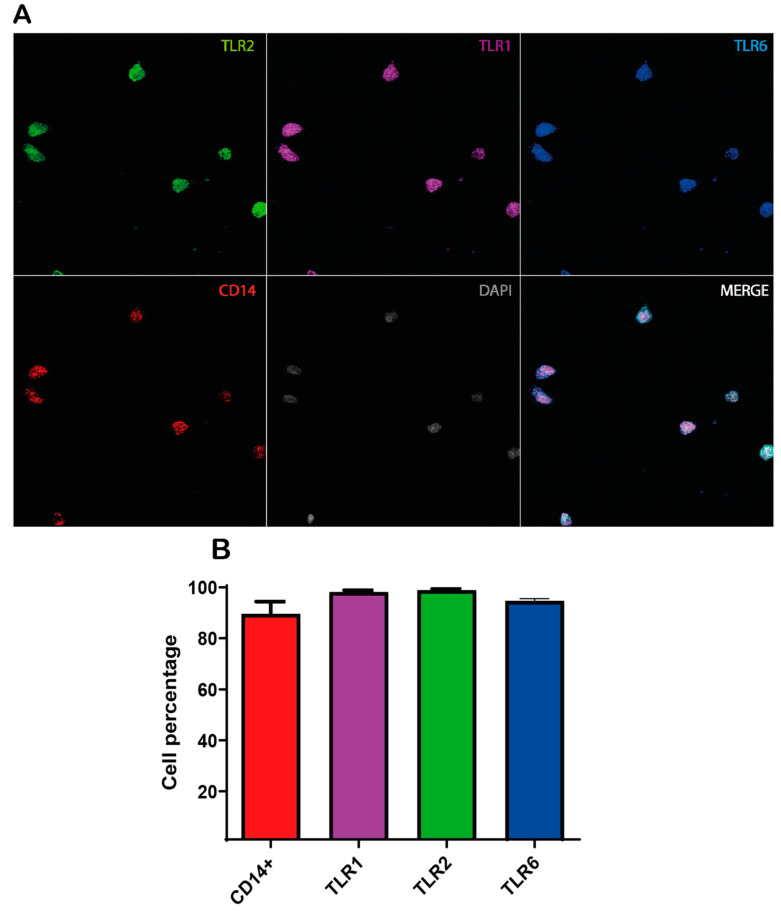
Immunofluorescent characterization of blood CD14+-monocytes isolation and detection of TLR1, TLR2, and TLR6 receptors. (**A**) Immunodetection of markers for DAPI (gray), CD14 (red), TLR1 (purple), TLR2 (green) and TLR6 (blue) were performed. (**B**) Graph of the percentage of immunoreactive cells to CD14 (87.6 ± 2.7), TLR1 (98.3 ± 2.1), TLR2 (99 ± 0.5) and TLR6 (89 ± 1.1).

Immunofluorescent staining for CD14 was conducted to evaluate the efficiency of an immunomagnetic positive selection of human peripheral blood monocytes using MACS^®^ technology (Figure 1A). CD14+ cells represented 87.5 ± 2.6% of the total nuclei count across five randomly selected fields. Among these CD14+ cells, the assessment of TLR immunoreactivity indicated that over 89% of the cells expressed TLR1, TLR2, and TLR6 (Figure 1B).

### 2.3. Gene Expression Analysis of TLR1, TLR2, and TLR6

**Figure 2:**
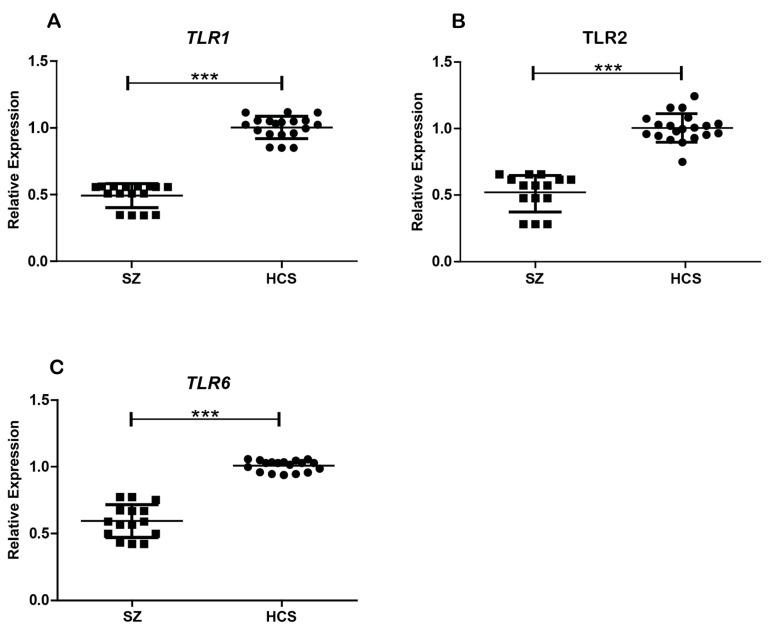
Relative expression of *TLR1*, *TLR2*, and *TLR6* genes in patients with SZ and HCSs. The relative expression of TLR was measured in peripheral blood monocytes via qPCR and normalized to the endogenous control gene. (**A**) *TLR1* mRNA expression in monocytes from patients with SZ and HCSs. (**B**) *TLR2* mRNA expression in monocytes from patients with SZ and HCSs. (**C**) *TLR6* mRNA expression in monocytes from patients with SZ and HCSs. Data are presented as the mean ± SEM, and significant differences between groups are indicated (*** *p* < 0.001).

Significant differences were observed in the gene expression levels of *TLR1* (*t* = 17.3, *p* < 0.0001), *TLR2* (*t* = 8.61, *p* < 0.0001), and *TLR6* (*t* = 13.8, *p* = 0.0001) between the total sample of SZ patients and HCSs (Figure 2). In monocytes from patients with SZ, the gene expression levels of all three TLRs were notably lower compared to those in HCSs.

**Figure 3:**
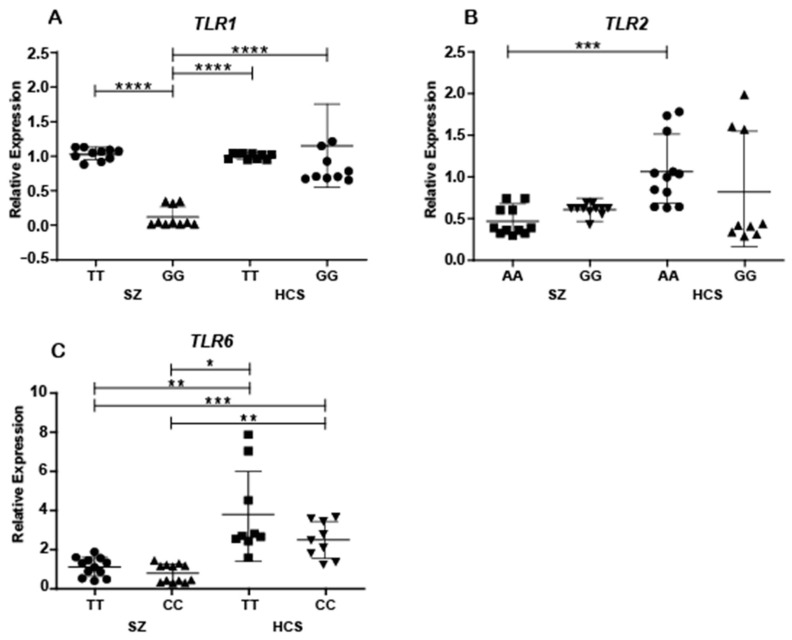
Expression levels of *TLR1*, *TLR2*, and *TLR6* mRNA in monocytes from patients with SZ and HCSs carrying the genotypes of interest. The relative expression of TLR was measured in peripheral blood monocytes via qPCR and normalized to the endogenous control gene. (**A**) *TLR1* mRNA expression in monocytes from SZ and HCSs carrying different genotypes for the rs4833093 variant (**** *p* = 0.0001). (**B**) *TLR2* mRNA expression in monocytes from patients with SZ and HCSs carrying different genotypes for the rs5743709 variant (*** *p* = 0.0006). (**C**) *TLR6* mRNA expression in monocytes from patients with SZ and HCSs carrying different genotypes for the rs3775073 variant (* *p* < 0.05, ** *p* < 0.01, *** *p* < 0.001).

The analysis of gene expression levels in patients with SZ revealed significant differences between the TT and GG genotypes of rs4833092/*TLR1* (*p* ≤ 0.0001). In contrast, no significant differences in gene expression were observed among HCSs when comparing the TT and CC genotypes. Additionally, patients with the GG genotype showed a marked decrease in gene expression compared to HCS carriers of both the TT genotype (*p* ≤ 0.0001) and the GG genotype (*p* ≤ 0.0001) (Figure 3A).

Significant differences in *TLR2* gene expression levels were observed between patients with SZ and HCSs carrying the AA genotype (*p* < 0.05). However, no significant differences were found in gene expression levels between SZ patients and HCSs carrying the GG genotype (Figure 3B).

A decrease in the expression levels of the *TLR6* gene was observed in patient carriers of the CC genotype of rs3775073/*TLR6* compared to HCS carriers of the CC and TT genotypes (*p* < 0.05). Additionally, significant differences were found between patients who were carriers of the CC genotype and HCSs carrying the TT genotype (*p* < 0.05). Finally, the comparison between patients with SZ and HCS carriers of TT genotype showed significant differences in gene expression levels (*p* < 0.05) (Figure 3C). In contrast, no significant differences in gene expression were observed among SZ when comparing carriers of the TT and CC genotypes.

### 2.4. Protein Expression Analysis of TLR1, TLR2, and TLR6

**Figure 4:**
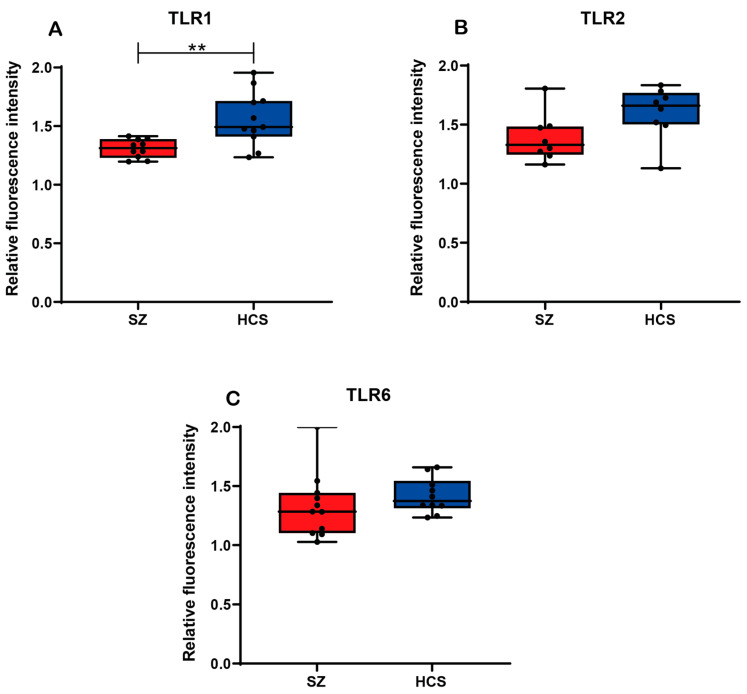
Relative fluorescence intensity of TLR1, TLR2, and TLR6 in monocytes from patients with SZ and HCSs. The relative protein expression of TLR was measured in peripheral blood monocytes via flow cytometry. (**A**) Comparison of protein expression of TLR1 in patients with SZ and HCSs. (**B**) Comparison of protein expression of TLR2 in patients with SZ and HCSs. (**C**) Comparison of protein expression of TLR6 in patients with SZ and HCSs. Data are presented as the mean ± SEM, and significant differences between groups are indicated (** *p* < 0.05).

To assess the levels of TLR1, TLR2, and TLR6 receptors in monocytes from patients with SZ and HCSs, an initial analysis was conducted without considering allele carriage (Figure 4). The protein expression analysis showed a significant decrease in TLR1 levels in monocytes from SZ patients compared to healthy controls (*p* < 0.05). However, no significant differences were found in the protein expression levels of TLR2 and TLR6 between the two groups (Figure 4).

**Figure 5:**
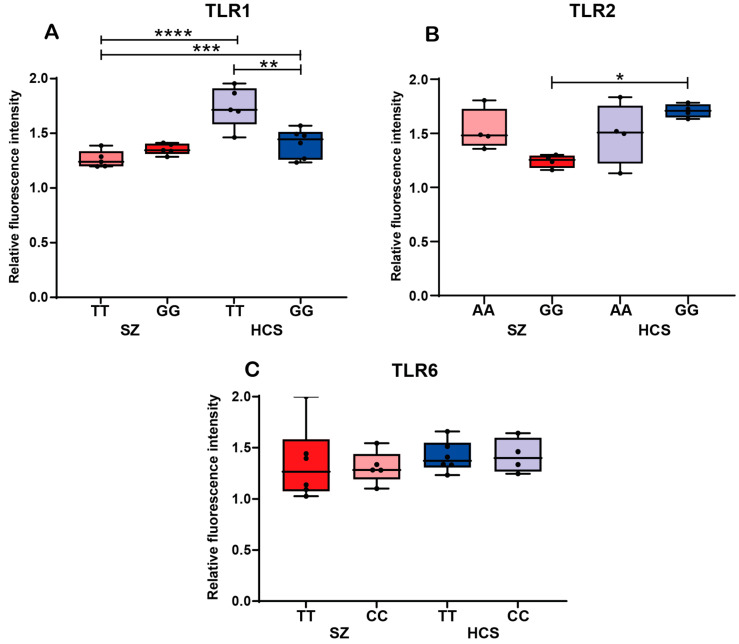
Protein expression of TLR1, TLR2, and TLR6 in monocytes of patients with schizophrenia and healthy controls carrying the genotypes of interest. The relative protein expression of TLR was measured in peripheral blood monocytes via flow cytometry. (**A**) TLR1 protein expression in monocytes from SZ and HCSs carrying different genotypes for the rs4833093 variant (** *p* = 0.0021, *** *p* = 0.0007, **** *p* < 0.0001). (**B**) TLR2 protein expression in monocytes from patients with SZ and HCSs carrying different genotypes for the rs5743709 variant (* *p* < 0.014). (**C**) TLR6 protein expression in monocytes from patients with SZ and HCSs carrying different genotypes for the rs3775073 variant.

Figure 5 illustrates the protein levels associated with TLR1, TLR2, and TLR6 genotypes in patients with SZ and HCSs. A significantly higher protein expression of TLR1 was observed in HCSs with the TT genotype compared to HCSs with the GG genotype of the rs4833093/*TLR1* variant (*p* = 0.0021). In contrast, patients carrying the TT genotype exhibited a decreased TLR1 protein expression compared to both HCSs with the TT genotype (*p* < 0.0001) and HCSs with the GG genotype (*p* = 0.0007).

TLR2 protein levels were significantly reduced in patients with the GG genotype compared to HCSs carrying the same rs5743709/*TLR2* genotype (*p* < 0.014) (Figure 5B). However, no significant differences in TLR2 protein expression were observed between the other genotype groups. In contrast, TLR6 protein expression analysis across different rs3775073/TLR6 genotypes did not reveal any significant differences between comparison groups (Figure 5C).

### 2.5. Functional Evaluation of TLR2/TLR6 in Human Peripheral Blood Monocytes

**Figure 6:**
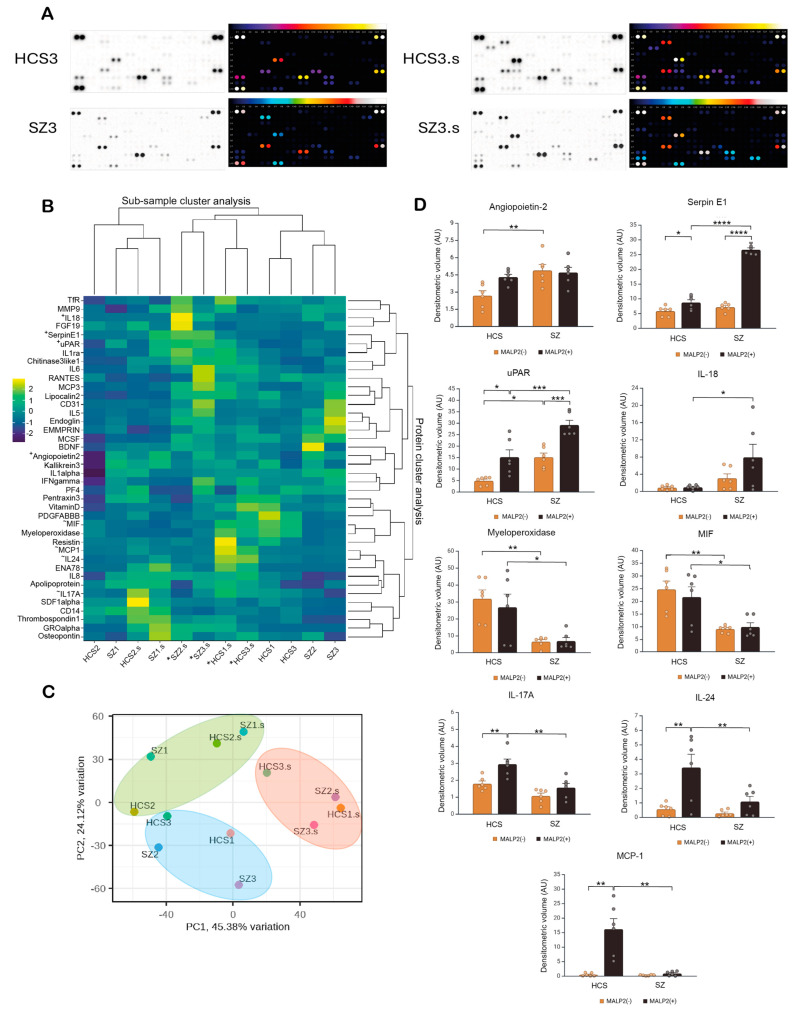
Exploratory data analysis of the secretory profile induced by TLR2/TLR6 ligand stimulation of CD14^(+)^-monocytes from patients with SZ and HCSs. Monocytes were incubated with 25 ng/mL MALP2 or without this ligand (MALP2-) in a serum-free medium for 24 h, and the cultured supernatants were collected for further proteomic immunoassays. (**A**) Representative images of the immunoreactive spots detected in the proteomic arrays and their corresponding representation as densitometric heatmaps. (**B**) Cluster analysis heatmap that shows the subsample cluster analysis and the protein clustering analysis. (**C**) Principal component analysis (PCS) biplot indicating the distribution of the subject samples in the first two components, which explains 69.5% of the total variance. (**D**) Graphic representation of the densitometric volume of the immunoreactive spots, showing significant differences between the SZ and HCS samples. Data represents the mean + SEM; statistical differences were assessed by two-way ANOVA and Tukey post hoc test for multiple comparisons, * *p* < 0.05; ** *p* < 0.01; *** *p* < 0.001; **** *p* < 0.0001. HCS (healthy control subject), SZ (schizophrenia patient), macrophage-activating lipopeptide 2 (MALP2).

A proteome array enabling the immunodetection of 105 analytes was used to identify the ligand-induced secretory profile of CD14+ monocytes from patients with SZ and HCSs (Figure 6A). Out of the 105 analytes, only 40 were detectable in the cell culture supernatants of both MALP2-stimulated (MALP2+) and unstimulated (MALP2-) monocytes. The subsample cluster analysis showed that MALP2- monocytes from two controls (HCS1, HCS3) and two patients with SZ (SZ2, SZ3) formed a cluster characterized by a generally low immunoreactive signal compared to their corresponding MALP2+ stimulated samples (HCS1.s, HCS3.s, SZ2.s, SZ3.s; samples marked with *) showing an increment in the signal (Figure 6B). Interestingly, another cluster with a consistently low immunoreactive secretory signal was formed by HCS2, HCS2.s, SZ1, and SZ1.s. Additionally, the protein cluster analysis revealed two main groups (Figure 6B). Interestingly, the proteins whose levels were significantly higher in patients with SZ (Angiopoietin 2, Serpin E1, uPAR, and IL-18; marked with ^+^) grouped together in the upper cluster analysis, whereas the proteins with significantly lower levels in SZ regarding the HCSs (myeloperoxidase, MIF, IL-17A, IL-24, and MCP-1; marked with ^~^) were grouped together in the lower cluster (Figure 6B).

The PCA (Figure 6C) showed us the same groups of HCS and SZ subjects that we obtained with the cluster analysis. Two principal components are represented in the biplot, explaining 69.5% of the total variance (PC1 45.38% and PC2 24.12%).

Panel D displays bar plots of the densitometric volume of immunoreactive spots for the nine proteins that showed significant differences between patients with SZ and HCSs. The immunoreactive signal for Angiopoietin 2 was higher in the supernatants of MALP2- monocytes from patients with SZ with no changes following MALP2 stimulation. Both serpin E1 and uPAR showed significantly higher densitometric volumes in MALP2+ cells, which were even higher in the supernatants of SZ regarding the HCS monocytes. Similarly, IL-18 levels were significantly elevated in MALP2+ monocytes from patients with SZ relative to HCSs under the same conditions. On the other hand, myeloperoxidase and MIF showed a significant reduction in SZ-derived monocyte supernatants regardless of MALP2 stimulation. Additionally, IL-17A, IL-24, and MCP-1 levels significantly increased in the supernatants of MALP2-stimulated HCS monocytes, but these signals were markedly reduced in SZ monocytes under the same conditions.

## 3. Discussion

Genetic association studies provide evidence of inflammatory-related genes in SZ [5,6,16]; however, the effect of the risk variants in the gene expression could provide additional information about their role in the development of SZ. The biological importance of these changes in the pathophysiology of SZ is unclear; therefore, we investigated the effect of three genetic variants previously associated with SZ, analyzing the gene expression levels in Mexican patients compared with a control group.

The analysis of clinical and demographic characteristics revealed that 96% of our patients were single. A lower marital rate has been observed among patients with this disorder, particularly among men [17]. Additionally, studies have reported that a stable marital status significantly enhances the quality of life in patients with schizophrenia [18]. It was also observed that patients with schizophrenia had a basic level of education and unemployment, which is a finding consistent with reports from other studies [19,20]. Schizophrenic patients tend to have lower educational attainment than the general population due to a combination of symptoms, including cognitive impairments such as memory, attention, and executive function deficits [21], which are characteristics that also impact getting a job [22]. Long-term employment is difficult for many individuals with schizophrenia due to employment discrimination and inadequate workplace support systems [23].

Our findings showed a decrease in the gene expression of *TLR1*, *TLR2*, and *TLR6* in patients compared to controls. Kozlowska et al. [15] reported alterations in the expression levels of the *TLR1*–*TLR9* genes in patients with SZ. However, this study was carried out in a European population, and they only evaluated changes in the gene expression of the TLRs between patients with SZ and healthy subjects.

To our knowledge, this is the first study aimed at identifying the role of genotype carriage in gene expression among patients with SZ. Our focus was on individuals who had homozygous copies of each of the polymorphic variants. *TLR1* observed a decrease in gene expression in patients with the GG genotype compared to those with the TT genotype and the control groups with either the TT or GG genotypes. These findings suggest that carrying the GG genotype may be associated with reduced *TLR1* gene expression in patients with SZ.

Regarding *TLR2*, genotype carriage analysis revealed lower gene expression in patients with the AA genotype compared to AA controls. This difference in expression may be influenced by other genetic factors or the underlying disease itself. Finally, the analysis of *TLR6* gene expression revealed significant differences between the study groups. Notably, patients with schizophrenia (SZ) exhibited markedly lower *TLR6* expression levels compared to healthy control subjects (HCSs) independent of genotype. However, no significant differences were detected between patients or controls with the different genotypes. This suggests a potential dysregulation of *TLR6* in individuals with schizophrenia, further supporting the hypothesis that *TLR6* may play a role in the immune-related mechanisms underlying the disorder [15]. These findings emphasize the need for further investigation into how genotype-specific variations in *TLR6* expression contribute to the pathophysiology of schizophrenia.

To investigate the potential impact of rs4833093 polymorphism in *TLR1*, we employed the JASPAR database to analyze DNA-binding motifs [24]. Our analysis revealed that rs4833093 is located within a binding site for several transcription factors, including One Cut Homeobox 1 (ONECUT1), which is associated with nervous system development [25]; Interferon Regulatory Factor 1 (IRF1), known for its role in immune response activation [26]; and Zinc Finger Protein 354A (ZNF354A), which facilitates DNA-binding transcription factor activity [27]. These findings suggest that the rs4833093/*TLR1* polymorphism could modify the binding affinity of these transcription factors, potentially altering *TLR1* gene expression levels.

It has been suggested that mRNA stability is affected by the number of optimal and non-optimal codons presented in a protein [28,29]. Genetic variants of *TLR2* and *TLR6* genes could be related to the mRNA stability, conferring a higher risk to developed SZ.

Regarding the analysis of TLR1 protein expression in the total sample, a lower level of the receptor was observed in patients with SZ compared to healthy controls. This finding aligns with the decreased gene expression observed in our previous analysis, suggesting a potential relationship between reduced *TLR1* gene expression and lower protein levels in patients with SZ. In contrast, no significant differences were found in the protein expression of TLR2 and TLR6 between patients and controls.

To our knowledge, this is the first study to investigate the role of polymorphic variants and protein expression in patients with SZ. The analysis of TLR1 protein expression, stratified by genotype, revealed a significant reduction in protein levels in patients with the TT genotype compared to TT and GG in controls. This finding suggests an association between TLR1 genotype carriage and SZ. However, no relation was observed between *TLR1* gene expression levels and protein expression, indicating the possible involvement of post-transcriptional and/or post-translational modifications that influence the regulation of gene and protein expression.

The relationship between gene expression and protein expression is inherently complex and subject to regulation at multiple stages of the gene expression process. The lack of correlation between mRNA and protein levels observed in this study can be attributed to several regulatory factors, including post-transcriptional, translational, and post-translational modifications.

Post-transcriptional regulation is a key determinant of protein levels [30]. After transcription, various regulatory mechanisms, such as microRNAs (miRNAs) and RNA-binding proteins, can bind to mRNA and modulate its stability or translational efficiency [31]. This can result in diminished protein production even in the presence of high mRNA levels. Furthermore, the alternative splicing of pre-mRNA can produce multiple isoforms with differing stability or functionality, further complicating the relationship between mRNA and protein levels [32]. The stability and degradation of mRNA are also important contributors to this discrepancy. mRNAs vary in their half-lives, with some undergoing rapid degradation while others persist longer within the cell, thereby affecting the total amount of protein synthesized. In addition, translational efficiency can be influenced by mRNA secondary structures, ribosomal availability, and other factors that impact the translation process [33].

Post-translational modifications (PTMs) also play a crucial role in regulating protein levels. Proteins may undergo a range of modifications after translation, such as phosphorylation, glycosylation, or ubiquitination, which can alter their stability, activity, or localization within the cell. These modifications may result in protein degradation or functional changes, reducing the detectable protein levels even if mRNA levels remain constant. Protein degradation pathways, particularly the ubiquitin–proteasome system, may further contribute to the observed discrepancies between mRNA and protein levels. Proteins marked for degradation may be rapidly removed from the cell, leading to reduced protein levels despite the continued presence of their corresponding mRNA transcripts [34].

In conclusion, the lack of correlation between mRNA and protein levels observed in this study likely reflects the influence of multiple regulatory layers, particularly post-transcriptional and post-translational modifications. These mechanisms highlight the complexity of gene expression, emphasizing that protein levels cannot always be directly inferred from mRNA levels. Therefore, it is essential to consider these regulatory mechanisms in future studies and investigate their role in the regulation of TLRs in patients with SZ.

The functional analysis of TLRs showed significant differences in expression profiles between patients with SZ and HCSs, which in turn may offer new insights into the underlying mechanisms of the disease and its response to immune-related receptor activation. Specifically, the hierarchical clustering of protein expression profiles revealed a distinction between the profiles of HCSs and patients with SZ. Notably, treatment with MALP2 seems to induce a marked shift in protein expression patterns in patients with SZ, suggesting a unique response to TLR activation, which may indicate an underlying immunological susceptibility.

Furthermore, principal component analysis (PCA) demonstrated a clear separation between the HCS and SZ groups, reflecting significant variability in protein expression profiles. The distinct clustering of HCS and SZ groups suggests that patients with SZ exhibit a unique proteomic profile. Moreover, MALP2 treatment intensifies the divergence between the groups, thereby indicating that this treatment exacerbates differences in protein expression, particularly in patients with SZ. However, partial overlap between the untreated HCS and SZ groups indicates that although there are baseline differences between the groups, MALP2 accentuates these distinctions. Additionally, the analysis of specific proteins further highlights the differences between the groups. In general, MALP2 treatment increases the levels of several inflammatory proteins, such as angiopoietin-2, Serpin E1, uPAR, and IL-18 in patients with SZ, suggesting an altered immune and inflammatory response.

The association of these molecules with inflammatory and tissue remodeling processes indicates that they may contribute to immune dysregulation in SZ, potentially influencing disease progression and symptomatology. For instance, angiopoietin-2, a key regulator of angiogenesis and vascular remodeling, normally stabilizes blood vessels, but its overexpression can lead to increased vascular permeability and inflammation [35]. In the context of SZ, elevated angiopoietin-2 may contribute to blood–brain barrier dysfunction, allowing inflammatory cells and mediators to enter the brain [36]. This disruption, in turn, could exacerbate oxidative stress and neuroinflammation, which are processes implicated in SZ pathogenesis [37,38,39,40].

Similarly, Serpin E1, a plasminogen activator inhibitor, plays a crucial role in regulating fibrinolysis and extracellular matrix remodeling. Its elevation in patients with SZ may suggest increased tissue remodeling activity and an altered fibrinolytic response [41]. Moreover, Serpin E1 overexpression has been linked to neurodegenerative diseases and is thought to promote chronic inflammation [42,43]. As a result, elevated Serpin E1 levels in patients with SZ could contribute to sustained inflammation and neuronal damage, thereby exacerbating cognitive deficits and functional impairments in these patients.

In addition, uPAR, a receptor that regulates extracellular matrix degradation and cellular signaling, plays a role in cell migration and inflammatory responses. Increased uPAR levels in patients with SZ may indicate heightened immune cell activation, which could lead to chronic inflammation [44]. Moreover, uPAR is also involved in cell adhesion and synaptic plasticity, which are critical factors for neuronal connectivity and development [45]. Thus, the dysregulation of uPAR may contribute to the structural brain abnormalities observed in SZ, negatively impacting synaptic function and neural connectivity [46,47].

Likewise, IL-18, a pro-inflammatory cytokine, plays a central role in both innate and adaptive immune responses. It is known to activate NK cells and T-helper cells as well as promote the production of other pro-inflammatory cytokines [48]. Indeed, elevated IL-18 levels in patients with SZ suggest persistent immune activation, potentially fostering an inflammatory environment in the central nervous system [49]. Furthermore, this cytokine has been associated with oxidative stress and cellular damage, contributing to neuronal deterioration and synaptic dysfunction. Additionally, IL-18 is linked to mood disorders and cognitive dysfunction, which are common symptoms in SZ [50,51].

Taken together, the elevated levels of angiopoietin-2, Serpin E1, uPAR, and IL-18 may promote chronic inflammation, blood–brain barrier dysfunction, and neuronal damage, contributing to the neuroinflammation and cognitive decline characteristic of SZ.

On the other hand, a decreased expression of myeloperoxidase (MPO), MIF, IL-17A, IL-24, and MCP-1 was observed in patients with SZ compared to HCSs. MPO, an enzyme produced by neutrophils and macrophages, generates reactive oxygen species (ROS), which are crucial for pathogen defense but can cause tissue damage if overproduced [52]. Thus, reduced MPO levels in SZ may indicate a diminished inflammatory response capacity, which could have counterproductive effects on innate immune function. Additionally, since oxidative stress is a key factor in SZ pathology, lower MPO activity may be related to an altered redox balance in the brain, affecting neuronal metabolism and synaptic function [53].

Likewise, MIF, a cytokine with both pro-inflammatory and immune regulatory properties, stimulates the release of other pro-inflammatory cytokines and promotes macrophage survival [54]. In SZ, reduced MIF levels may reflect impaired macrophage activation and a diminished capacity to mount adequate inflammatory responses. This reduction could result in a less reactive cerebral environment, affecting processes such as neurogenesis and synaptic plasticity, which are essential for maintaining neuronal health [55].

Moreover, IL-17A, a cytokine produced by T-helper (Th17) cells, is essential for defense against bacterial and fungal infections and plays a key role in autoimmune inflammation [56]. Therefore, the reduction in IL-17A in patients with SZ may indicate decreased Th17 cell activation, suggesting possible immunosuppression or the dysregulation of specific inflammatory responses [57]. The decrease in IL-17A could also affect the blood–brain barrier, as this cytokine facilitates immune cell migration into the brain [58], potentially limiting the brain’s ability to respond to pathogenic challenges and contributing to neuroinflammatory dysfunction.

In addition, IL-24, a cytokine involved in apoptosis regulation and the inflammatory response, plays a role in tissue repair and remodeling and has immunomodulatory properties [59]. Reduced IL-24 expression in patients with SZ may indicate a decreased ability to repair damage and respond appropriately to inflammation. This reduction, in turn, could be related to the neurodegeneration observed in SZ, as IL-24 contributes to neuronal protection and regeneration [60]. Consequently, a lack of IL-24 could exacerbate the effects of other pro-inflammatory pathways, facilitating neuronal damage and altering synaptic functions.

Finally, MCP-1, a chemokine critical for recruiting monocytes and other immune cells to sites of inflammation, is also reduced in patients with SZ. This suggests that reduced immune cell recruitment to the brain may impair the central nervous system’s ability to respond to pathological processes [61]. Lower MCP-1 activity could indicate an insufficient immune response, reducing the brain’s capacity to clear damaged cells and respond to chronic inflammation [62]. Moreover, MCP-1 is associated with neurogenesis and synaptic plasticity, and its reduction may negatively impact the formation of new neuronal connections and the maintenance of the neuronal network [61,62,63].

Altogether, the decreased levels of MPO, MIF, IL-17A, IL-24, and MCP-1 in patients with SZ may impair the brain’s ability to respond to damage and maintain neuronal homeostasis, thus exacerbating neurodegeneration and synaptic plasticity issues commonly seen in SZ. These findings underscore the importance of considering immune dysregulation in SZ and suggest that modulation of these inflammatory pathways could represent a potential therapeutic approach.

In conclusion, the results suggest that MALP2 has a more pronounced impact on protein expression in patients with SZ than in healthy controls. This may indicate that TLR activation contributes to SZ pathophysiology by exacerbating an altered inflammatory state. Thus, the modulation of these receptors could represent a potential therapeutic target in the treatment of SZ, and these findings highlight the importance of further studying the role of TLRs in this disease.

## 4. Materials and Methods

### 4.1. Sample

The sample comprised twenty-four patients diagnosed with schizophrenia according to the Diagnostic and Statistical Manual of Mental Disorders 5 (DSM-5) criteria using the Spanish version of the Mini International Neuropsychiatric Interview (MINI-5).

Inclusion criteria were patients who were 18 years or older, had no chronic immunologic diseases, and did not have a first- or second-degree family member participating in the study. The patients were recruited from the Schizophrenia Clinic of the Instituto Nacional de Psiquiatría Ramón de la Fuente Muñiz in Mexico City. The exclusion criteria were active infection at the time of blood sampling, patients diagnosed with an autoimmune or chronic inflammatory disease, and patients who have been on non-steroidal or steroidal anti-inflammatory drugs for at least a week. The control group consisted of twenty-four participants who were not diagnosed with a psychiatric disorder, without a family history of psychiatric disorders, and did not have a first- or second-degree family member participating in the study. Exclusion criteria included subjects with an active infection at the time of blood sampling, subjects diagnosed with an autoimmune or chronic inflammatory disease, and subjects in treatment with non-steroidal or steroidal anti-inflammatory drugs for at least one week.

The forty-eight subjects consisted of 8 homozygous individuals (4 patients with schizophrenia and 4 controls) for the two alleles of each gene variant. All participants were Mexican with a family background generation of at least three generations born in Mexico.

The Alcohol, Smoking, and Substance Involvement Screening Test (ASSIST), developed by the World Health Organization ASSIST Working Group [64], was used to evaluate both the lifetime and recent (past 3 months) use of 10 substances as well as symptoms of abuse and dependence in patients and controls. All substances analyzed by the ASSIST were assessed in this study. However, among the substances evaluated, alcohol and tobacco were the only ones reported as consumed by patients within the past 3 months.

### 4.2. Isolation of Peripheral Blood Mononuclear Cells (PBMCs)

Venous blood samples (15 mL) were obtained in Vacutainer™ tubes with EDTA. All tubes were identified with a numerical code to protect the identity of the participants. The isolation of PBMCs was performed by the density gradient centrifugation method according to Fuss et al. [65]. Briefly, blood samples were centrifuged for 15 min at 2000× *g* at room temperature (RT) to obtain a leukocyte-enriched buffy coat. The buffy coat was collected and resuspended with 10 mL of Hank’s Balanced Saline Solution (HBSS). Leukocyte-enriched suspension was slowly layered over Ficoll-Hypaque^®^ solution (3 mL of Ficoll-Hypaque^®^ per 9 mL cell suspension) and centrifuged for 30 min at 900× *g*, RT. The PBMCs-enriched interphase was transferred to another conic tube, washed twice, and centrifuged for 10 min at 600× *g*, RT, for platelet depletion. Cell count was performed with a hemocytometer, and cell viability was determined by trypan blue exclusion.

### 4.3. Positive Selection of CD14^+^ Cells with MACS^®^ Technology

CD14^+^ monocytes were enriched from human PBMCs using a positive selection protocol with the MACS^®^ system according to the manufacturer’s instructions (Miltenyi Biotec, Bergisch Gladbach, Germany). Briefly, PBMCs were incubated with CD14+ magnetic beads (Cat. 130117337; 20 μL/10^7^ cells, 2–4 °C for 15 min); then, while the magnetic column was attached to the magnet, the cell suspension passed through the LS column (130042401, Miltenyi Biotec, Bergisch Gladbach, Germany) for monocyte sorting by positive selection. The monocyte-depleted cell suspension was used to perform the genotyping of the variants of interest. Then, the column was detached from the magnet, and the CD14^+^-monocytes-enriched cell suspension was eluted with the plunger. Cell count was performed with a hemocytometer, and cell viability was determined by trypan blue exclusion.

### 4.4. Immunofluorescent Detection of CD14, TLR1, TLR2 and TLR6

Immediately after MACS^®^ column elution, the cell suspension enriched in CD14^+^-monocytes was washed twice with HBSS and finally resuspended in RPMI 1640 supplemented with 10% heat-inactivated fetal bovine serum, 1× GlutaMAX, 1× penicillin–streptomycin, and then plated into 12 mm round glass coverslips at 25,000 cells/well in 4-well culture plates. Cells were incubated overnight at 37 °C, 5% CO_2_, with a humid atmosphere. After incubation, cells were fixed with 4% paraformaldehyde for 10 min and washed twice with TBS. Blocking was performed with 10% donkey serum, 0.3 M glycine, and 0.05% Tween 20 in TBS for 30 min. TLRs were detected by indirect fluorescence using the following primary antibodies: rabbit anti-TLR1 (Novus Biologicals, Centennial, CO, USA. NB100-56563), mouse anti-TLR2 (Novus Biologicals, Centennial, CO, USA. NB100-56722) and rabbit anti-TLR6 (Novus Biologicals, Centennial, CO, USA. NBP1-76664), all diluted 1:200 in 1% donkey serum, 0.05% Tween20 in TBS, overnight, at 4 °C in a humid chamber protected from light. After washing 4 times with TTBS (0.1%), cells were incubated with the corresponding secondary antibodies, i.e., Alexa Fluor^®^ 647-conjugated donkey anti-rabbit IgG (Abcam ab150075, Cambridge, UK; dilution 1:1000) and Alexa Fluor^®^ 488-conjugated donkey anti-mouse IgG (Jackson ImmunoResearch 715-545-150, West Grove, PA, USA; dilution 1:800) in TTBS for 1 h at room temperature and protected from light. CD14 was detected by direct immunofluorescence using an R-PE-conjugated anti-human CD14 (Life Technologies MHCD1404, Thermo Fisher, ScientificWaltham, MA, USA), which was diluted 1:150. After extensive washing, 1 µM DAPI was added for 10 min for nuclei counterstaining, washed with deionized water, and finally, coverslips were mounted with CitiFluor^®^ (Hatfield, PA, USA) for further image acquisition. Images were acquired with an LSM-900 confocal microscope with Airyscan (ZEISS) and analyzed with FIJI Image J version 2.9.0 (NIH Image J, Bethesda, MD, USA).

### 4.5. Flow Cytometry Analysis

To evaluate the protein expression of TLR1, 2, and 6 in monocytes from patients and healthy controls, an analysis by flow cytometry was performed. First, 4.5 × 10^6^ CD14^+^-monocytes were fixed with 4% paraformaldehyde and resuspended in PBS. Subsequently, indirect immunofluorescence was performed for either TLR1/AF647 (NB100-56563; Novus Biologicals, Centennial, CO, USA), TLR2/AF488 (NB100-56722; Novus Biologicals, Centennial, CO, USA), or TLR6/AF647 (NBP1-76664; NBP1-76664) for 30 min. CD14/PE antibody (Life Technologies, Carlsbad, CA, USA, MHCD1404) was incubated for 30 min. Cells were analyzed using the Attune flow cytometer (Thermo Fisher, ScientificWaltham, MA, USA), and data were analyzed with the WinList™ software package version 10.1.

### 4.6. RNA Extraction, cDNA Obtention, and Quantitative Analysis by PCR

Total RNA was extracted from monocytes using TRIzol™ (Roche; Germany) reagent, following the manufacturer’s instructions. The first-strand cDNA was synthesized with SuperScript™ IV VILO™ Master Mix (Thermo Fisher Scientific, Waltham, MA, USA) in three technical replicates per sample using 500 ng of total RNA. The expression assay was assessed with TaqMan probes for *TLR1* (Hs00413978_m1), *TLR2* (Hs02621280_s1), *TLR6* (Hs00271977_s1) and *ACTB* (Hs99999903_m1) as housekeeping gene, using the ABI Prism ^®^7500 Sequence Detection System according to the manufacturer’s protocols (Applied Biosystems Inc.; Foster City, CA, USA). The PCR reaction was performed in a 10 µL reaction volume, consisting of 500 ng of cDNA, 1× TaqMan Universal Master Mix II and 0.5× of TaqMan Gene Expression Assay. cDNA was amplified for 40 cycles at 95 °C for 15 s and 60 °C for 1 min.

### 4.7. Assessment of TLRs Function: Analysis of Ligand-Induced Secretory Profile

To conduct an exploratory analysis of TLR function, we randomly selected a subsample consisting of three patients and three controls for proteomic analysis of the ligand-stimulated secreted molecules. Two culture dishes per individual were prepared, each containing 2.25 × 10^6^ monocytes. The cells were cultured in RPMI 1640 medium supplemented with 10% fetal bovine serum, 1× GlutaMAX, and 1× penicillin–streptomycin. The cultures were incubated overnight at 37 °C in a humidified atmosphere with 5% CO_2_. After total culture medium change for serum-free medium, one of the dishes was stimulated with 25 ng/mL of macrophage-activating lipopeptide 2 (MALP-2) (Novus Biologicals, NBP2-26219, Centennial, CO, USA), a specific ligand for the heterodimer TLR2/TLR6, while the other dish served as an unstimulated control sample. After incubation for 24 h, supernatants were collected and stored at −20 °C until further analysis. The total protein in the supernatants was determined with the BCA Protein Assay kit (Pierce, Appleton, WI, USA) as recommended by the manufacturer. The culture supernatants (500 µg of protein per sample) were assayed to immunodetect the monocyte-secreted factors using the Proteome Profiler™ Human XL Cytokine Array Kit (R&D Systems, Minneapolis, MN, USA), following the protocol provided by the manufacturer. The volumetric densitometry of the immunoreactive spots was determined with the FIJI/ImageJ software version 2.9.0 (Bethesda, MD, USA). The data were normalized by subtracting the densitometric volume of the negative control spots and expressed as a percentage of the densitometric volume of the positive control spots of the immunoarray.

### 4.8. Statistical Analyses

The demographic and clinical characteristics of patients and controls were analyzed using Student’s *t*-test for continuous variables such as age, body mass index, and blood pressure. Additionally, the chi-square test was employed for categorical variables, including sex, marital status, level of education, employment status, and substance use. An additional analysis was performed to assess the potential confounding effects for evaluating their association between tobacco and alcohol use in cases and controls. Also, we evaluated the variables’ confusers, such as tobacco and alcohol use, age of onset, age of diagnosis, and pharmacological treatments in cases stratified by the carriage of the alleles. No significant differences were observed, confirming that these variables do not act as confounding factors in the study.

The gene expression analysis was performed using GraphPad Prism software version 5.0.0 [66]. Cycle threshold values were averaged, and relative *TLR1, TLR2*, and *TLR6* gene expression levels were determined using the comparative cycle threshold method [67]. The expression data were normalized using a Q-Q plot analysis to assess the distribution of the data. Differences between the *TLR1*, *TLR2*, and *TLR6* expression of patients and controls were calculated using a *t*-test. All the results were normalized to *ACTB.* Changes in gene expression in relationship to the allelic carriage were analyzed using ANOVA and Tukey’s multiple comparison post hoc test. The amount of relative fluorescence of TLR1, TLR2, and TLR6 from the flow cytometry analysis between patients and controls was calculated. ANOVA and Tukey’s multiple comparison post hoc tests were analyzed to evaluate differences in relative fluorescence in relation to the allelic carriage in patients and controls.

A heatmap and a PCA were created for the exploratory data analysis of the secretory profiles. The first approach to the experimental values was a heatmap scaled by row (z scale for each variable) and non-directed classification dendrograms. Each column represented a different subject with or without treatment. The other form of visualization was with the biplot derived from the PCA method. With this approach, the first two components were used to explain our data variability. The heatmap was assessed using the library heatmaply ver. 1.4.0 and the PCA approach with the library PCA tools ver. 2.16.0. all within the R version 4.4.1 software package.

## 5. Conclusions

This study provides new insights into the role of *TLR1, TLR2*, and *TLR6* genetic variants in the regulation of gene and protein expression in Mexican patients with SZ. Our findings demonstrate a significant reduction in the gene expression of *TLR1, TLR2* and *TLR6* in patients with SZ compared to HCSs, suggesting that TLRs play an important role in the pathophysiology of SZ. Specifically, the rs4833093 polymorphism in *TLR1* might alter transcription factor binding, potentially affecting gene expression and contributing to the immune dysregulation observed in SZ. At the protein level, TLR1 expression was reduced in patients with SZ, particularly among those carrying the TT genotype. However, this reduction in protein levels did not correlate directly with mRNA expression, highlighting the complex regulatory mechanisms involved, including post-transcriptional and post-translational modifications. TLR2 and TLR6 protein expression did not show significant differences between patients and controls, underscoring the need for further investigation into the influence of these genes on SZ pathogenesis.

Additionally, the analysis of inflammatory protein profiles revealed altered immune responses in SZ patients supporting the hypothesis of immune dysfunction in this disorder. Treatment with MALP2 exacerbated these differences, suggesting that TLR activation may contribute to the unique immunological landscape of SZ.

In conclusion, our findings emphasize the importance of *TLR* gene variants in modulating immune responses in SZ, providing a potential avenue for future therapeutic interventions targeting immune dysregulation in this disorder. Further studies are needed to explore the precise mechanisms through which these TLRs contribute to the etiology of SZ.

## Figures and Tables

**Table 1:** Demographic and clinical characteristics of the sample.

Characteristic	Controls (n = 24)	Patients (n = 24)	Statistics
Age, mean (SD), years	36.4 (5.3)	41.7 (3.2)	t = 1.63, *p* = 0.108
Sex, n (%)			
Male	13 (59.1)	15 (62.5)	X^2^ = 0.05, *p* = 0.812
Female	9 (40.91)	9 (37.5)	
Marital status, n (%)			
Married/cohabiting	10 (41.7)	1 (4)	X^2^ = 9.97, *p* = 0.0016
Single	14 (58.3)	24 (96)	
Level of education, n (%), years			
Elementary	2 (8)	11 (46)	X^2^ = 16.1, *p* = 0.0003
Secondary	6 (25)	10 (41)	
Higher	16 (57)	3 (13)	
Employment status, n (%)			
Yes	24 (100)	13 (54.1)	X^2^ = 14.2, *p* = 0.0002
No	0 (0)	11 (45.9)	
Tobacco use, n (%)	15 (62.5)	14 (58.3)	X^2^ = 0.08, *p* = 0.767
Alcohol use, n (%)	14 (66.7)	19 (79.2)	X^2^ = 0.89, *p* = 0.344
Systolic blood pressure, mean (SD), mmHg	120 (11.1)	117 (14.2)	*t* = 0.69, *p* = 0.492
Diastolic blood pressure, mean (SD), mmHg	73.8 (10.3)	73.5 (10.2)	*t* = 0.098, *p* = 0.921
Body mass index, mean (SD), kg/m^2^	27.5 (7.1)	29.3 (6.4)	*t* = 0.918, *p* = 0.363
Age of onset, mean (SD), years	-	23.2 (8.4)	
Age of diagnosis, mean (SD), years	-	26.1 (8.5)	
Second-generation antipsychotic treatment, n (%)	-	15 (62)	
First-generation antipsychotic treatment, n (%)	-	11 (46)	
Antidepressant treatment, n (%)	-	13 (54)	

## Data Availability

The raw data supporting the conclusions of this article will be made available by the authors on request.
